# Abnormal Interactions of the Salience Network, Central Executive Network, and Default-Mode Network in Patients With Different Cognitive Impairment Loads Caused by Leukoaraiosis

**DOI:** 10.3389/fncir.2019.00042

**Published:** 2019-06-18

**Authors:** Hongyan Chen, Yuexiu Li, Qi Liu, Qingli Shi, Jingfang Wang, Huicong Shen, Xuzhu Chen, Jun Ma, Lin Ai, Yu Mei Zhang

**Affiliations:** ^1^Department of Radiology, Beijing Tiantan Hospital, Capital Medical University, Beijing, China; ^2^Department of Rehabilitation Medicine, Beijing Tiantan Hospital, Capital Medical University, Beijing, China; ^3^Center of Stroke, National Clinical Research Center for Neurological Diseases, Beijing, China; ^4^Beijing Institute for Brain Disorders, Beijing, China; ^5^Beijing Key Laboratory of Translational Medicine for Cerebrovascular Disease, Beijing, China; ^6^Beijing Key Laboratory of Central Nervous System Injury, Beijing, China; ^7^Department of Neurology, Beijing Tiantan Hospital, Capital Medical University, Beijing, China; ^8^Department of Neurology, Beijing Pinggu Hospital, Beijing, China; ^9^Department of Neurology, General Hospital of The Yang Tze River Shipping, Wuhan Brain Hospital, Wuhan, China; ^10^Department of Nuclear Medicine, Beijing Tiantan Hospital, Capital Medical University, Beijing, China

**Keywords:** leukoaraiosis, resting-state MRI, functional connectivity, salience network, central executive network, default-mode network

## Abstract

Leukoaraiosis (LA) is associated with cognitive impairment in the older people which can be demonstrated in functional connectivity (FC) based on resting-state functional magnetic resonance imaging (rs-fMRI). This study is to explore the FC changes in LA patients with different cognitive status by three network models. Fifty-three patients with LA were divided into three groups: the normal cognition (LA-NC; *n* = 14, six males), mild cognitive impairment (LA-MCI; *n* = 27, 13 males), and vascular dementia (LA-VD; *n* = 12, six males), according to the Mini Mental State Exam (MMSE) and Clinical Dementia Rating (CDR). The three groups and 30 matched healthy controls (HCs; 11 males) underwent rs-fMRI. The data of rs-fMRI were analyzed by independent components analysis (ICA) and region of interest (ROI) analysis by the REST toolbox. Then the FC was respectively analyzed by the default-mode network (DMN), salience networks (SNs) and the central executive network (CEN) with their results compared among the different groups. For inter-brain network analysis, there were negative FC between the SN and DMN in LA groups, and the FC decreased when compared with HC group. While there were enhanced inter-brain network FC between the SN and CEN as well as within the SN. The FC in patients with LA can be detected by different network models of rs-fMRI. The multi-model analysis is helpful for the further understanding of the cognitive changes in those patients.

## Introduction

Leukoaraiosis (LA), also called white matter hyperintensities (WMH), was described as multifocal or diffuse periventricular or subcortical hyperintensity lesions of varying sizes (Hachinski et al., 1987). LA is found in 39% population and in 96% population above 60 years (Longstreth et al., 1996). The progression of LA is associated with cognitive impairments (Schmidt et al., 2007; O’Sullivan, 2008; Brickman et al., 2011; Chen et al., 2016).

Resting-state functional magnetic resonance imaging (rs-fMRI), including functional connectivity (FC), has been used in cognitive impairments (He et al., 2014; Cheng et al., 2017). FC has been identified as some robust intrinsic connectivity networks (ICNs), such as the default-mode network (DMN), central executive network (CEN) and salience network (SN; Seeley et al., 2007; Menon and Uddin, 2010; Menon, 2011; Chand et al., 2017). A triple network model made by DMN, CEN and SN has shown an explanatory power for psychiatric and neurological diseases (Seeley et al., 2007; Menon, 2011; Chand and Dhamala, 2016).

FC may be a potential predictor of cognitive impairments in patients with LA (Reijmer et al., 2015; Cheng et al., 2017; Li et al., 2017). Previous study has shown that the DMN, SN and CEN interact closely to control cognitive processes. Reijmer et al. (2015) reported the alternation of the DMN [the posterior cingulate cortex (PCC) and medial prefrontal cortex (MPFC)] in patients with WMH. Cheng et al. (2017) found an increased FC between the right insular region (an important area in SN) and the right superior orbital frontal gyrus and between the right calcarine cortex and the left PHG in LA patients. Chand et al.’s (2017) study showed the SN could modulate the interaction between DMN and CEN in healthy elderly controls but this modulation was disrupted in mild cognitive impairment MCI. However, the interactions of these brain networks associated with cognitive impairment loads in patients with LA are still undescribed. Therefore, our study aimed to investigate the connectivity patterns of DMN, SN and CEN by using rs-fMRI data in LA patients with different cognitive impairment.

## Materials and Methods

### Participants

**Figure 1:**
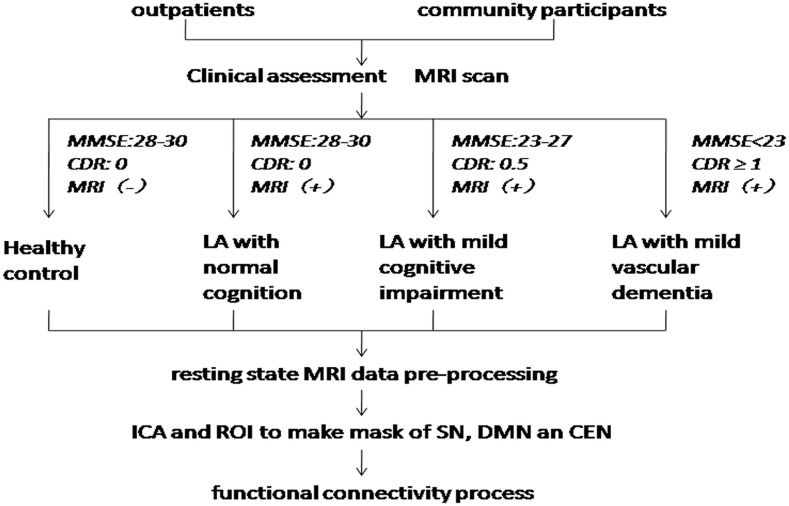
Flow chart. MMSE, Mini Mental State Exam; CDR, Clinical Dementia Rating; LA, leukoaraiosis; ICA, independent components analysis; ROI, region of interest; SN, salience networks; DMN, default-mode network; CEN, central executive network.

A total of 53 LA patients and 30 healthy control (HC) subjects in this study were consecutively recruited from the Beijing Tiantan Hospital. The inclusion criteria for the LA patients were as above: (1) age ≥50 years; (2) patients have LA lesions on MRI images according to the revised version of the scale of Fazekas; and (3) right-handed. The exclusion criteria were as above: (1) MRI contraindications; (2) severe systemic diseases; (3) related neurological diseases, such as epilepsy, traumatic brain injury, multiple sclerosis; (4) leukoencephalopathy of non-vascular origin; (5) dementia of non-vascular origin; and (6) inability to complete cognitive test and MRI exam. All of the LA patients have diffuse or confluent white matter hyperintensity lesions in periventricular or subcortical white matter on T2-weighted image (T2WI) and fluid-attenuated inversion recovery (FLAIR) MRI. HC subjects have the matched age, gender and education year with LA. All experimental protocols in our study were approved by the institutional review board of Beijing Tiantan Hospital, and all subjects signed informed consent forms. The flow chart of this study has been showed in Figure 1.

### Clinical Assessment

The cognitive function of the four groups was evaluated by Mini Mental State Exam (MMSE) and their clinical diseases state was judged by the clinical dementia rating scale (CDR). All clinical scales were evaluated by two experienced neurologists.

Based on the MRI images and the scores of the MMSE, they were divided into four groups: LA patients with normal cognition (LA-NC; *n* = 14, MMSE scores range 28–30), LA patients with MCI (LA-MCI; *n* = 27, MMSE scores range 23–27), and LA patients with vascular dementia (VD; LA-VD; *n* = 12, MMSE scores less than 23). HC group (*n* = 30, MMSE scores range 28–30).

### Image Acquisition

MR images were acquired using a 3T SIEMENS TIM whole-body MR system. We used a T2WI sequence with the following scan parameters: repetition time (TR) = 4,500 ms, echo time (TE) = 84 ms, flip angle (FA) = 120°, matrix = 256 × 256, field of view (FOV) = 220 × 220 mm^2^, slice thickness = 5 mm, and slice gap = 1 mm, number of slices = 24. We used a T2WI-FLAIR sequence with the following scan parameters: TR = 11,000 ms, TE = 140 ms, FA = 90°, matrix = 256 × 256, FOV = 220 × 220 mm^2^, slice thickness = 3.5 mm, and slice gap = 1.0 mm. The 3D MPRAGE sequence with the following scan parameters: TR = 2,300 ms, TE = 3.28 ms, FOV = 256 mm × 256 mm^2^, matrix = 256 × 256, flip angle 9°, in plane resolution = 1 mm × 1 mm, slice thickness = 1 mm. rs-fMRI data were acquired using an echo planar imaging (EPI) sequence with the following scan parameters: TR = 2,000 ms, TE = 30 ms, flip angle (FA) = 90°, matrix = 64 × 64, FOV = 256 × 256 mm^2^, slice thickness = 3.7 mm, and slice gap = 0 mm, number of slices 32. During the fMRI scans, all subjects were asked to keep their eyes closed, stay as motionless as possible, think of nothing in particular, and not fall asleep during the scan.

### Pre-processing of Resting-State Data

All of the fMRI data were analyzed using SPM8^1^. The pre-processing steps include: slice-timing realignment and adjustment, motion correction, co-registration, normalization, spatial smoothing, delinearization and bandpass filtering. The first five volumes were discarded to eliminate T1 relaxation effects. Next, the head motion parameters of each volume were estimated and saved, and each volume was realigned to the mean map of the whole volumes to correct for geometric displacements using a six-parameter rigid-body transformation. Five subjects were excluded from further analysis because they had maximum displacements (>2 mm) in one or more of the orthogonal directions (x, y, z) or a maximum rotation (x, y, z) >2.0°. The data were spatially normalized to the standard EPI template and re-sampled to 2 × 2 × 2 mm^3^. The normalized data were smoothed using a 6-mm full width at half maximum.

### Definition of the SN Mask by the ICA Method and Intra-Brain-Network FC

**Figure 2:**
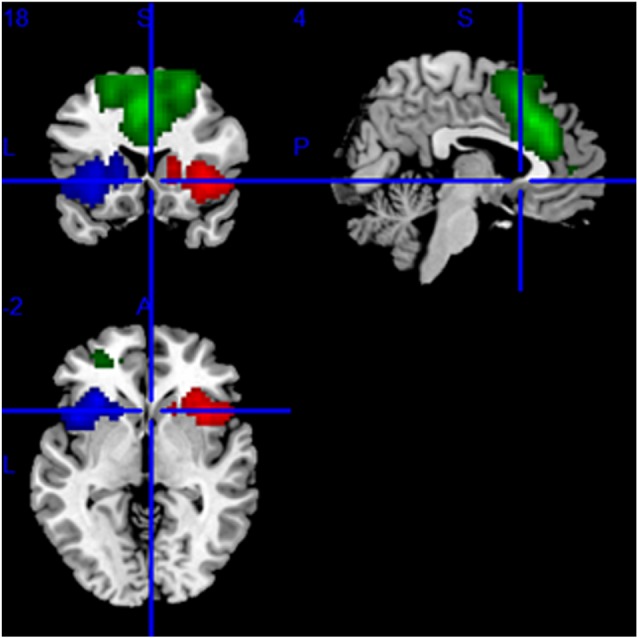
The mask of the SN, mainly including the bilateral frontoinsular cortex (FIC) and anterior cingulate cortex (ACC). The red area is the right, the blue area is the left FIC, and the green area is ACC.

We applied group independent component analysis (gICA) to rs-MRI data with the Infomax algorithm implemented in Matlab (Calhoun et al., 2001) in order to identify the regions of interest (ROIs) for the SN. To ensure the stability of the ROIs, we performed gICA100 times. Twenty-five aggregate independent components (ICs) were identified using the GIFT toolbox^2^, in which the number of components was determined by the minimum description length criterion. All aggregate ICs were visually inspected, and the IC representing SN was selected (Seeley et al., 2007). Then, the specify IC of all subjects regardless of group was entered into a random-effect one-sample *t*-test. We used a threshold of *P* < 0.001 [Family Wise Error (FWE) correction] to define the SN mask. The result was displayed on a brain surface template using the REST slice viewer^3^. Based on this result, the mask SN was made using MRIcro^4^ and displayed on the brain surface template (Figure 2).

In order to obtain the intra-brain-network FC of each group, we repeated the same group ICA test for each group. ICs representing the SN of each patient were selected and entered into a random-effect one-sample *t*-test. The result of the one-sample *t*-test was used to obtain the intra-brain-network FC for each ROI in the SN.

### Definition of the DMN and CEN Masks by ROI and Intra-Brain-Network FC

**Figure 3:**
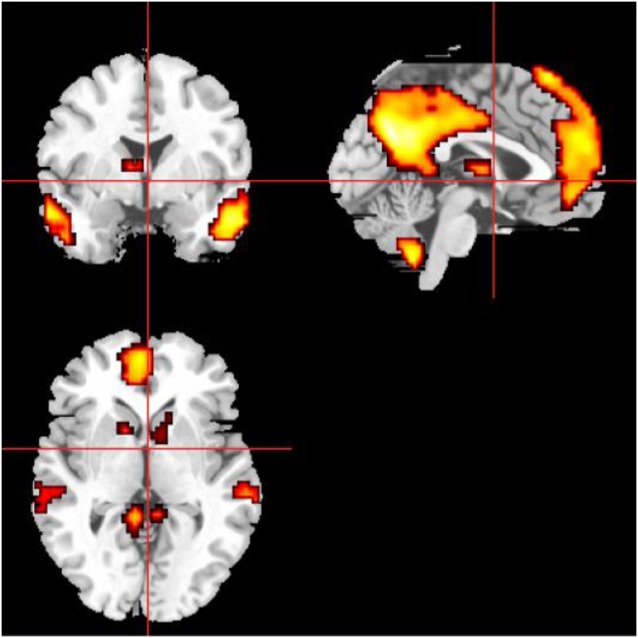
The mask of the DMN, mainly including medial prefrontal cortex (MPFC), ACC, posterior cingulate cortex (PCC), and precuneus.

**Figure 4:**
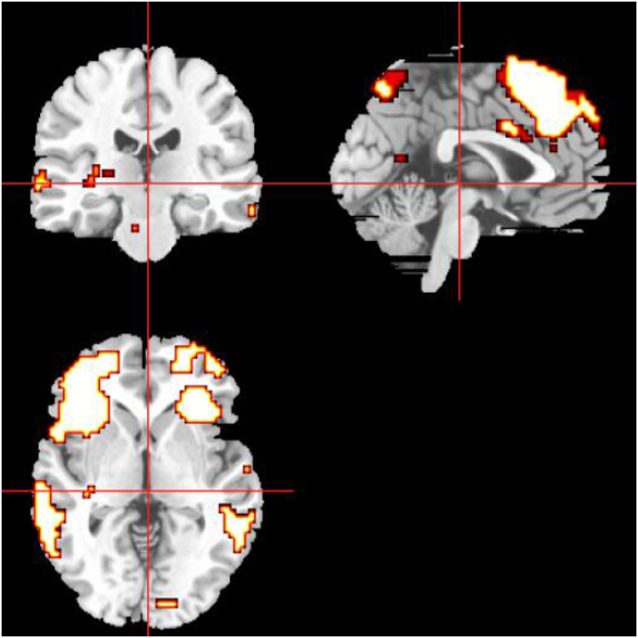
The mask of the CEN, mainly including the ACC and bilateral dorsolateral PFC (dlPFC).

The definitions of the ROIs of the DMN and CEN were based on a previous study (Duan et al., 2012). For the DMN, we defined three ROIs, including the ventromedial PFC (vmPFC, MNI coordinates: −2, 54, −6), left PCC (MNI coordinates: −6, −48, 32), and right PCC (MNI coordinates: 10, −52, 28). For the CEN, we defined four ROIs, including the left dorsolateral PFC (dlPFC; MNI coordinates: −48, 34, 34), right dlPFC (MNI coordinates: 48, 40, 30), left PPC (MNI coordinates: −36, −44, 46), and right PPC (MNI coordinates: 42, −42, 48). Then, a sphere mask of 10 mm in radius centered at each peak voxel was defined and the averaged time series for each of the defined ROIs was extracted. Then we used the averaged time series extracted from the SN template as the starting point, the FC measurement was performed for each voxel of the whole brain. Conjunction analysis was used to define the brain areas that were correlated with all of the ROIs of the specific functional networks. Then we used the FWE method with a threshold of *P* < 0.05 after multiple comparison corrections. Finally, the ROIs were selected as the masks capturing the key nodes within the DMN and CEN. The masks of DMN and CEN were shown on the MRIcrobrain surface template (Figures 3, 4).

### SN-Anchored FC Analysis

First, the right frontoinsular cortex (FIC) was selected as the seed region to calculate inter-brain-network FCs with the CEN and DMN because of its reported role in switching between the CEN and the DMN (Sridharan et al., 2008). We extracted the averaged time series of the right FIC to calculate its FC with each voxel across the whole brain. With the application of the DMN and CEN masks, the mean FCs of the core regions within the DMN and CEN were measured. ANOVA was used to determine the differences for both inter-brain-network and intra-brain-network FCs among these four groups. Between-group differences of the intra-brain-network FCs between the SN and DMN as well as between the SN and CEN were also compared.

### Statistical Analyses

We used one-way ANOVA to compare the clinical features (age, education year, MMSE scores, CDR scores) and the FC across the four groups. Bonferroni correction and least significant difference (LSD) were used to *post hoc* comparisons.

## Results

### Participant Characteristics

In the present study, a total of 53 LA patients and 30 HCs were included in all the analyses. The demographic and behavioral statistics for the four groups are presented in Table 1. There were no significant differences in age or education among the four groups (*P* > 0.1).

**Table 1:** Demographic and behavioral statistics for the four groups.

Items	HC	LA-NC	LA-MCI	LA-VD
Age (years)	54.3 ± 9.6	60.1 ± 5.3	62.0 ± 11.4	59.9 ± 12.8
Gender (M/F)	11/19	6/8	13/14	6/6
Education (years)	13.3 ± 3.0	12.0 ± 2.8	11.0 ± 3.2	11.1 ± 3.1
MMSE	28.9 ± 1.5	29.1 ± 1.0	27.2 ± 2.4	23.3 ± 3.4
CDR	0	0	0.5	≥1

*CDR, clinical dementia rating; F, female; HC, healthy controls; LA, leukoaraiosis; M, male; MCI, mild cognitive impairment; MMSE, Mini Mental State Exam; VD, vascular dementia*.

### The FC of Whole Brain Based on Right FIC

**Figure 5:**
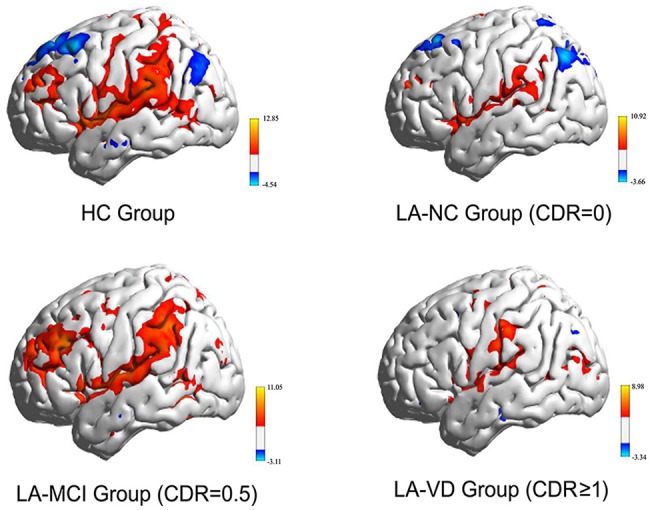
The functional connectivity (FC) of the whole brain of each of the four study groups. The FC of the three LA groups is markedly weaker than that of the healthy control (HC) group. CDR, clinical dementia rating; MCI, mild cognitive impairment; VD, vascular dementia.

Figure 5 showed the whole brain FC of the three LA groups and HC group based on the right FIC as the seed point. All the FC of LA groups were lower than HC, the next order was the LA-MCI, the LA-NC and the LA-VD.

### The Relationship Between SN and DMN

The interaction between the SN and DMN was negatively correlated. The one-way ANOVA was used for inter-networks of SN and DMN.

**Figure 6:**
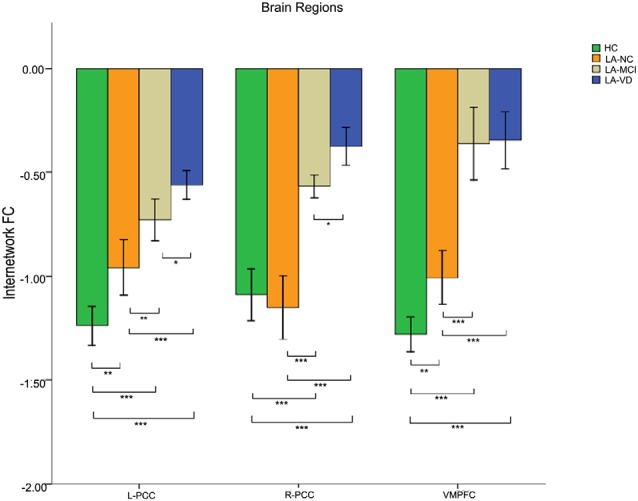
The FC between anatomical brain regions in the DMN and SN. There was negative correlation between DMN and SN activity. The first group of bars is the left PCC, the second group of bars is the right PCC, the third group of bars is the ventromedial PFC (vmPFC). The overall connectivity trend from high to low was HC group, LA-normal cognition (NC) group, LA-MCI group, and LA-VD. Green bar, HC; yellow bar, LA-NC; gray bar, LA-MCI; and blue bar, LA-VD. *P < 0.05, LSD corrected; **P < 0.05, Bonferroni corrected; ***P < 0.001, Bonferroni corrected. Error bars represent standard error. Error bar: 95% confidence intervals (CIs).

**Figure 7:**
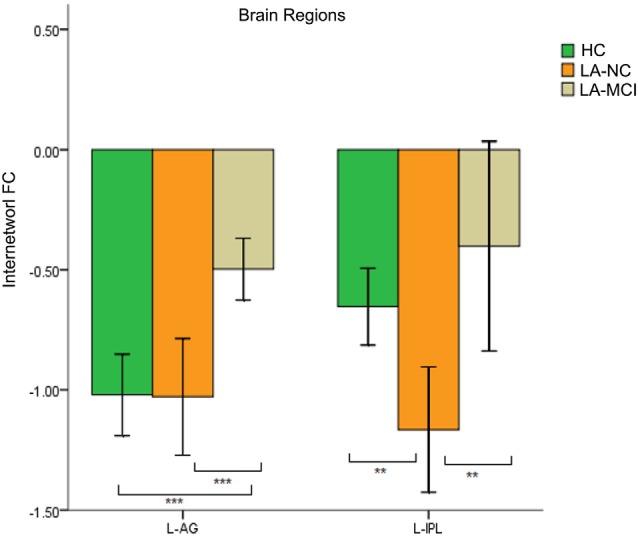
The FC between anatomical brain regions in the DMN and SN. Within the left angular gyrus (AG) and left inferior parietal lobe (IPL), the number of negatively activated points in the LA-VD group was too small to be counted. The first group of bars is the left AG, the second group of bars is the left IPL. Green bar, HC; yellow bar, LA-NC; and gray bar, LA-MCI. **P < 0.05, Bonferroni corrected; ***P < 0.001, Bonferroni corrected. Error bars represent standard error. Error bar: 95% CI.

We found differences in the bilateral PCC and bilateral vmPFC among the four groups, and we also found differences in the left AG and left inferior parietal lobe (IPL) among the HC group, LA-NC group and LA-MCI group (Figure 6). Within the left AG and left IPL, the number of negatively activated points in the LA-VD group was too small to be counted, so we only compared the FC of the HC group, the LA-NC group and the LA-MCI group (Figure 7).

This result showed that for the FC between the SN and DMN, the HC group has the highest connection, followed by LA-NC, LA-MCI and LA-VD.

### The Relationship Between SN and CEN

**Figure 8:**
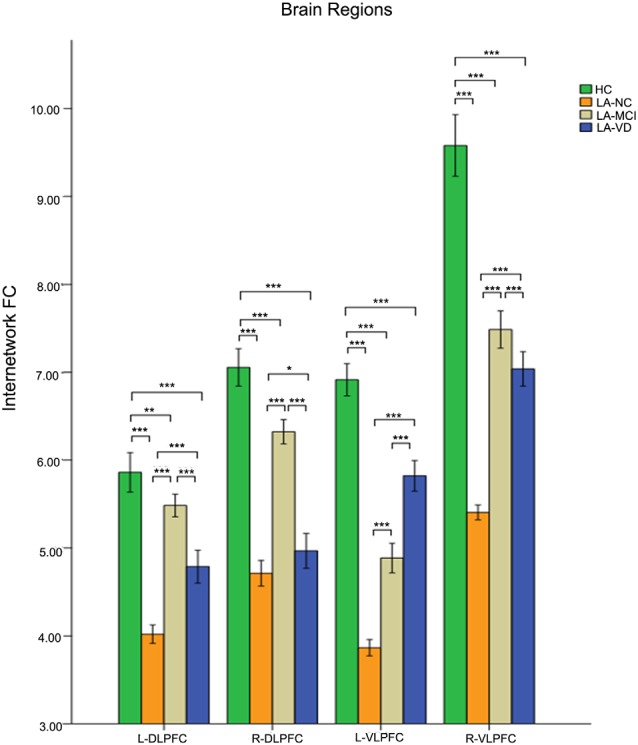
The FC between anatomical brain regions in the CEN and SN. There was a positive correlation between CEN and SN activity. The first group of bars is the left dlPFC, the second group of bars is the right dlPFC, the third group of bars is the left ventrolateral PFC (vlPFC), and the fourth group of bars is the right vlPFC. The overall connectivity trend from high to low was HC group, LA-MCI group, LA-VD group, and LA-NC group. Green bar, HC; yellow bar, LA-NC; gray bar, LA-MCI; blue bar, LA-VD. *P < 0.05, LSD corrected; **P < 0.05, Bonferroni corrected; ***P < 0.001, Bonferroni corrected. Error bars represent standard error. Error bar: 95% CI.

**Figure 9:**
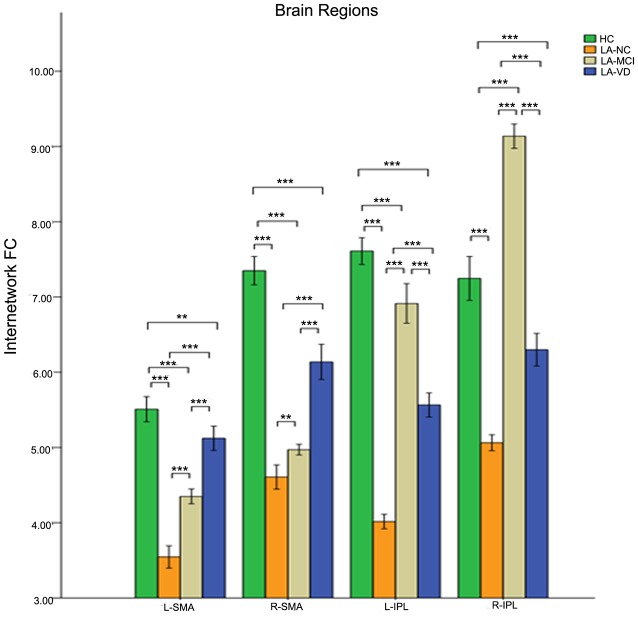
The FC between anatomical brain regions in the CEN and SN. There was a positive correlation between CEN and SN activity. The first group of bars is the left supplementary motor area (SMA), the second group of bars is the right SMA, the third group of bars is the left IPL, the fourth group of bars is the right IPL. The overall connectivity trend from high to low was HC group, LA-MCI group, LA-VD group and LA-NC group. Green bar, HC; yellow bar, LA-NC; gray bar, LA-MCI; blue bar, LA-VD. **P < 0.05, Bonferroni corrected; ***P < 0.001, Bonferroni corrected. Error bars represent standard error. Error bar: 95% CI.

The interaction between the SN and CEN was positively correlated. The overall connectivity trend was different from DMN, the HC group also has the highest FC, but its next order was LA-MCI, LA-VD and LA-NC. We found significant connectivity differences in the bilateral dlPFC, bilateral ventrolateral PFC (vlPFC), bilateral supplementary motor area (SMA), and bilateral IPL among the four groups (Figures 8, 9).

### The Relationship Within the SN

**Figure 10:**
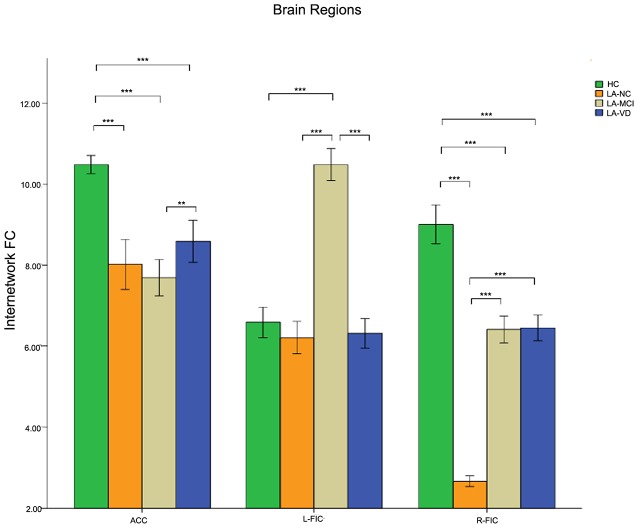
The result of the intra-SN FC. There were positive correlations in neural activity. The first group of bars is the ACC, the second group of bars is the right FIC. The overall connectivity trend from high to low was HC group, LA-VD/MCI group, and LA-NC group. Green bar, HC; yellow bar, LA-NC; gray bar, LA-MCI; blue bar, LA-VD. **P < 0.05, Bonferroni corrected; ***P < 0.001, Bonferroni corrected. Error bars represent standard error. Error bar: 95% CI.

Nearly all the three components of SN showed significant differences across groups when used the one-way ANOVA, including the bilateral FICs and anterior cingulate cortex (ACC). The *post hoc* comparison of the intra-brain-network FC of the SN was positively correlated in terms of neural activity. The connectivity of HC also was the highest one, and the LA-VD/ LA-MCI followed HC, the least one was LA-NC. We found significant FC in the ACC and the right FIC among the four groups (Figure 10).

## Discussion

In this study, we examined the FC of the SN, CEN, and DMN in LA patients with different cognitive impairment loads and HC participants. We found that different LA groups with different cognitive status presented differently in the ICNs compared to the HC group. There were four findings revealed in our study. First, the negative correlations between the SN and DMN were diminished as the cognitive impairment loads increased. As the load of cognitive, the decline of FC in LA groups was intensified. The FC between the SN and DMN provided a characterization of the cognitive load impairment of the LA. Second, with the increase in the severity of cognitive impairment, the positive correlation between the SN and CEN was increased. The FC between the SN and CEN provided a representation of LA’s cognitive load compensation. Last, as the severity of LA cognitive impairment increased, the positive correlation within the SN was increasing. The intra-SN connection provided a representation of the cognitive burden compensation for LA.

In our study, the LA patients demonstrated decreased functional connectivity between the DMN and the right FIC in SN, especially in the bilateral PCC and vmPFC, consistent with previous studies (Menon and Uddin, 2010; Li et al., 2015; Reijmer et al., 2015; Atwi et al., 2018; Wang et al., 2019). It may indicate that LA leads to the decrease of FC between the SN and DMN, and the damage of connectivity between the DMN and SN. Wang et al. (2019) found that the PCC, precuneus and right inferior temporal gyrus showed significantly decreased ALFF values in LA patients. The researchers thought that the executive functional impairment of LA may be caused by the disruptions in the afferents of PCC. Although we used different method, we got the similar result. Atwi et al. (2018) found that older adults with WMH had lower activation in fronto-temporal and parietal cortices compared to the healthy older adults, so they got the conclusion WMH may contribute to the dysfunction of brain networks. Our result is consistent with this result. Reijmer et al. (2015) combined the structural and functional brain connectivity in WMH patients, and they found that the PCC and MPFC of the DMN were altered, as well as a significant correlation between the microstructural properties of the cingulum bundle and MPFC-PCC FC in patients with low WMH load. This decreased structural connectivity could explain the change in FC, and this result could explain our study. But more evidence of structural and FC is needed to ensure the physiological basis of LA patient’s cognitive changes.

Our study showed that the CEN and the right FIC in SN were positively correlative, and the FC was decreased between the CEN and SN in LA patients regardless of the cognitive status. This may be explained by Wiggins research, since the LA contributed to the speeded and mental manipulation of executive function (Wiggins et al., 2018). Interestingly, the LA-MCI and LA-VD groups in our study showed higher FC between the CEN and SN, especially in the bilateral dlPFC, ventrolateral PFC, bilateral SMA, and IPL. This may due to the changes in functional compensation in LA patients with cognitive dysfunction (Lockhart et al., 2015; Gold et al., 2017).

Our study also revealed decreased FC in the intra-SN at the ACC and right FIC in LA patients ignorance of their cognitive status. As an important part of the SN, FIC is a critical switcher between the CEN and DMN (He et al., 2014). He et al. (2014) found that the bilateral FIC showed decreased intra-SN FC in the AD group. The decrease of FC may indicate that the intra-SN connection has been damaged in LA groups, and it may be caused by LA. Furthermore, the LA-MCI and LA-VD group showed significant increase in intra-SN FC at the right FIC when compared with the LA-NC group. This mechanism may play a compensatory role in LA patients with MCI and VD, improving the efficiency of conversion. Since the FC in LA patients has changed and suggests a compensatory mechanism (Cheng et al., 2017) the intra-SN connection may provide a representation of the cognitive burden compensation for LA.

There are limitations of our study. First, the sample is not large enough. Second, this study did not contain a correlation analysis of the specific relationships among different cognitive functions and the three ICNs. Third, we did not analyze the relationship between the FC and the neuropsychological battery.

## Conclusion

In our study, we evaluated the rs-fMRI by a triple network model in LA patients with different cognitive status. The multi-model analysis is helpful for further understanding of the cognitive changes in LA patients.

## Data Availability

All datasets generated for this study are included in the manuscript.

## Ethics Statement

This study was carried out in accordance with the recommendations of the institutional review board of Beijing Tiantan Hospital with written informed consent from all subjects. All subjects gave written informed consent in accordance with the Declaration of Helsinki. The protocol was approved by the institutional review board of Beijing Tiantan Hospital.

## Author Contributions

HC, QL, QS, YL, and JW performed the experiment. HC analyzed image data, drafted the manuscript and performed the statistical results. QL, QS, YL, and JW collected the data involved in the study. YZ designed the study. YZ, HS, JM, LA and XC gave critical comments on the manuscript.

## Conflict of Interest Statement

The authors declare that the research was conducted in the absence of any commercial or financial relationships that could be construed as a potential conflict of interest.

## Footnotes

^1^http://www.fil.ion.ucl.ac.uk/spm/software/spm8

^2^http://icatb.sourceforge.net/groupica.htm

^3^http://www.restfmri.net/forum/REST

^4^http://mccauslandcenter.sc.edu/CRNL
